# Incidence rate of occult lymph node metastasis in clinical T_1−2_N_0_M_0_ small cell lung cancer patients and radiomic prediction based on contrast-enhanced CT imaging: a multicenter study: Original research

**DOI:** 10.1186/s12931-024-02852-9

**Published:** 2024-05-29

**Authors:** Xu Jiang, Chao Luo, Xin Peng, Jing Zhang, Lin Yang, Li-Zhi Liu, Yan-Fen Cui, Meng-Wen Liu, Lei Miao, Jiu-Ming Jiang, Jia-Liang Ren, Xiao-Tang Yang, Meng Li, Li Zhang

**Affiliations:** 1 Department of Diagnostic Radiology,National Cancer Center/National Clinical Research Center for Cancer/Cancer Hospital, Chinese Academy of Medical Sciences and Peking Union Medical College, Beijing, 100021 China; 2 Department of Radiology, Sun Yat-sen University Cancer Center, State Key Laboratory of Oncology in South China, Collaborative Innovation Center for Cancer Medicine, Guangzhou, 510060 China; 3 Department of Radiology, The Third People’s Hospital of Chengdu, Chengdu, 610031 China; 4 Department of Radiology, Shanxi Cancer Hospital, Shanxi Medical University, Taiyuan, 030013 China; 5 Department of Pathology, National Clinical Research Center for Cancer/Cancer Hospital, National Cancer Center, Chinese Academy of Medical Sciences and Peking Union Medical College, Beijing, 100021 China; 6 Department of Radiology, The First Hospital of China Medical University, Shenyang, 110001 China; 7 Department of Pharmaceuticals Diagnostics, GE HealthCare, Beijing, 100176 China

**Keywords:** Small-cell lung cancer, Occult lymph node metastases, Contrast-enhanced computed tomography, Radiomics

## Abstract

**Background:**

This study aimed to explore the incidence of occult lymph node metastasis (OLM) in clinical T_1 − 2_N_0_M_0_ (cT_1 − 2_N_0_M_0_) small cell lung cancer (SCLC) patients and develop machine learning prediction models using preoperative intratumoral and peritumoral contrast-enhanced CT-based radiomic data.

**Methods:**

By conducting a retrospective analysis involving 242 eligible patients from 4 centeres, we determined the incidence of OLM in cT_1 − 2_N_0_M_0_ SCLC patients. For each lesion, two ROIs were defined using the gross tumour volume (GTV) and peritumoral volume 15 mm around the tumour (PTV). By extracting a comprehensive set of 1595 enhanced CT-based radiomic features individually from the GTV and PTV, five models were constucted and we rigorously evaluated the model performance using various metrics, including the area under the curve (AUC), accuracy, sensitivity, specificity, calibration curve, and decision curve analysis (DCA). For enhanced clinical applicability, we formulated a nomogram that integrates clinical parameters and the rad_score (GTV and PTV).

**Results:**

The initial investigation revealed a 33.9% OLM positivity rate in cT_1 − 2_N_0_M_0_ SCLC patients. Our combined model, which incorporates three radiomic features from the GTV and PTV, along with two clinical parameters (smoking status and shape), exhibited robust predictive capabilities. With a peak AUC value of 0.772 in the external validation cohort, the model outperformed the alternative models. The nomogram significantly enhanced diagnostic precision for radiologists and added substantial value to the clinical decision-making process for cT_1 − 2_N_0_M_0_ SCLC patients.

**Conclusions:**

The incidence of OLM in SCLC patients surpassed that in non-small cell lung cancer patients. The combined model demonstrated a notable generalization effect, effectively distinguishing between positive and negative OLMs in a noninvasive manner, thereby guiding individualized clinical decisions for patients with cT_1 − 2_N_0_M_0_ SCLC.

**Supplementary Information:**

The online version contains supplementary material available at 10.1186/s12931-024-02852-9.

## Introduction

Lung cancer is the most common cause of cancer-related death worldwide and accounts for approximately 18.0% of all such deaths [1]. Lung cancer can be divided into small cell lung cancer (SCLC) and non-small cell lung cancer (NSCLC) based on histological subtype, and SCLC accounts for approximately 15% of all lung cancer cases [2]. SCLC is a high-grade neuroendocrine carcinoma with an exceptionally poor prognosis and an overall 5-year survival rate of only 7% [3]. Previous studies have shown that concurrent chemoradiotherapy (CCRT) has been the standard treatment for SCLC since the early 1990s [4, 5]. With the widespread use of CT, the number of early peripheral SCLC tumours has increased [6–8]. Recent attention has shifted towards surgical intervention, revealing promising 5-year survival rates of up to 50% for pathological T_1 − 2_N_0_M_0_ SCLC patients [9–11]. Hence, the National Comprehensive Cancer Network guidelines recommend surgery as the primary treatment modality for pathological T_1 − 2_N_0_M_0_ SCLC [12, 13].

However, in clinical practice, while imaging is effective in determining the T_1 − 2_ stage, defining N_0_ is challenging because surgical lymph node dissection often yields positive results when imaging does not indicate lymph node metastases [14–16]. Occult lymph node metastasis (OLM) refers to the situation in which lymph node metastasis is not detected by presurgical imaging (mainly CT) but is confirmed by postoperative pathology [17–19]. Preoperative imaging examinations rely mainly on CT to diagnose lymph node metastasis, but many OLMs are missed, resulting in ineffective surgery. For cT_1 − 2_N_0_M_0_ SCLC patients, the presence or absence of OLM determines whether the patient is able to undergo surgery. Thoracoscopic biopsy is the “gold standard” for detecting the status of chest lymph nodes, but this is an invasive examination method that may lead to a series of complications, such as bleeding, infection, and pneumothorax. Therefore, identifying new and valuable noninvasive imaging methods for predicting OLM in cT_1 − 2_N_0_M_0_ SCLC patients is necessary.

In recent years, radiomics has emerged as a prominent area of research, allowing for the high-throughput extraction of extensive data from medical images [20]. This approach enables the analysis of high-level and quantitative image features, providing a profound reflection of the spatial heterogeneity within tumour tissues [20]. Previous studies have successfully developed models for predicting OLM in NSCLC patients based on radiomic features of primary lesions (including lesions with ground-glass density and solid density), demonstrating robust predictive performance [21–24]. Additionally, peritumoral radiomics has been proven equally predictive [25, 26]. Furthermore, the literature has focused predominantly on OLM in NSCLC [26–29], with limited studies exploring the incidence rate of OLM in SCLC.

Consequently, this study focused on cT_1 − 2_N_0_M_0_ SCLC patients to investigate the incidence rate of OLM in this clinical population and developed predictive models for OLM that integrate clinical parameters and intratumoral and peritumoral contrast-enhanced CT radiomics.

## Materials and methods

### Patient selection

The institutional review boards approved this retrospective study, and the requirement for written informed consent was waived. The histopathology of the tumours was defined according to the 2015 World Health Organization definition [30], and clinical and pathological staging was based on the 8th edition of the TNM classification [31]. This study retrospectively reviewed 242 patients with SCLC confirmed by postoperative pathology from four centeres between January 2014 and September 2022. The inclusion criteria were as follows: (1) underwent resection of the primary lesion and systematic lymph node dissection; (2) underwent preoperative enhanced CT; and (3) had a clinical stage before surgery of T_1 − 2_N_0_M_0_. Additionally, all patients had solitary pulmonary nodules in clinical stage T_1 ~ 2_ based on enhanced CT imaging and no enlarged lymph nodes (i.e., short diameter of LN ≤ 10.0 mm on CT imaging). The exclusion criteria were as follows: (1) patients who received radiotherapy, chemotherapy, or other treatments for SCLC before surgery; (2) had an interval between CT examination and surgery of more than 2 weeks; (3) had thin-layer images (with a slice thickness less than or equal to 1.25 mm) missing; and (4) had severe CT artifacts and poor image quality. For patients with multiple lesions, only SCLC lesions with conclusive pathological results were included. The patient’s lymph node metastasis was obtained from the postoperative pathology report and reconfirmed by a senior pathology professor in the Department of Pathology.

**Fig. 1:**
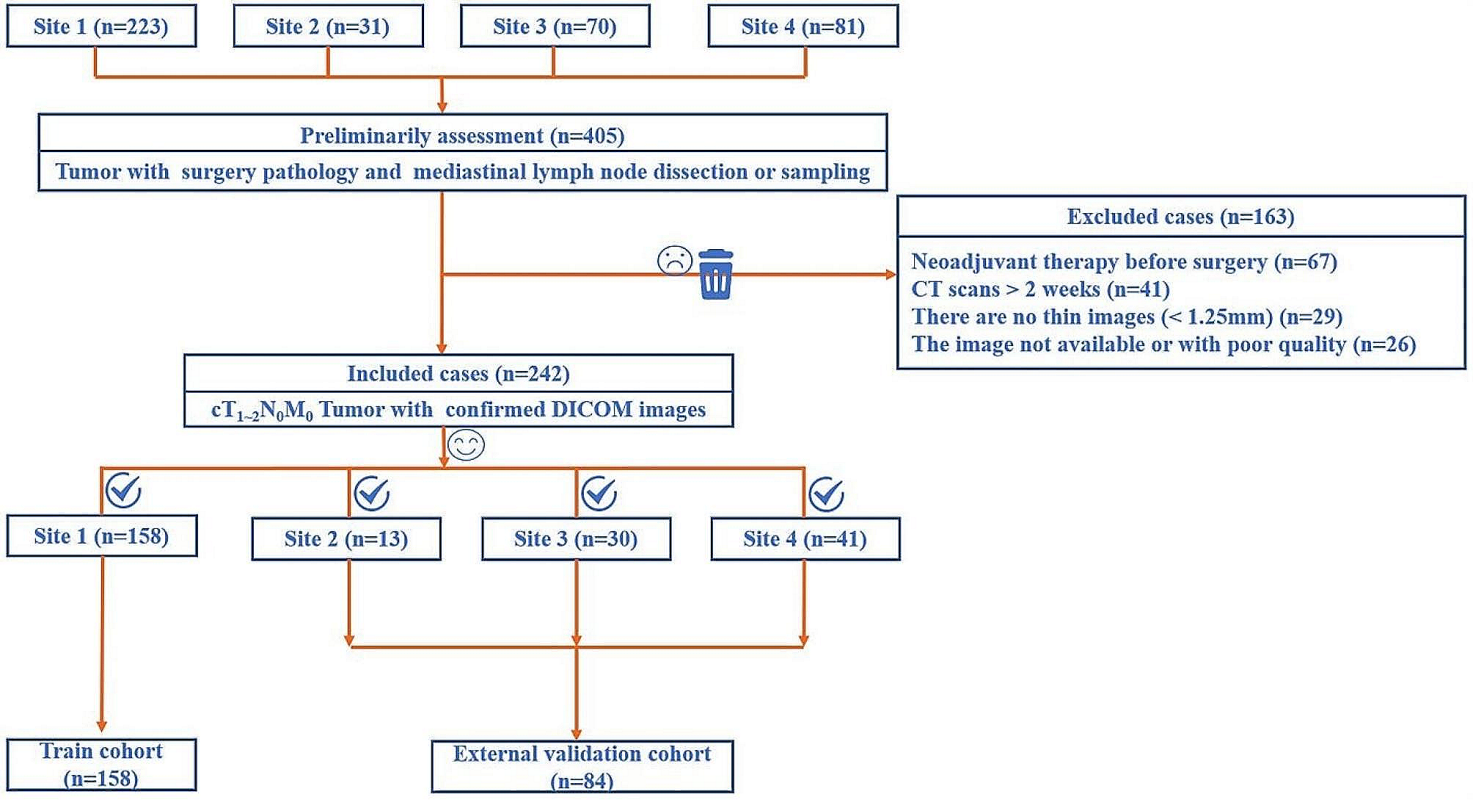
Flow diagrams showing the pathways associated with patient inclusion and exclusion. SCLC = small-cell lung cancer. DICOM = Digital Imaging and Communications in Medicine

All patients from center 1 were allocated to the training cohort, and patients from center 2, 3 and 4 composed the external validation cohort (Fig. 1).

### CT scanning and semantic CT features

All enrolled patients in the four hospitals underwent a similar scan setup but with different systems and parameters (Appendix E1). The definitions and evaluation criteria for clinical parameters are described in Appendix E2. Two radiologists, each with 2 years of experience in lung imaging and blinded to the clinical and pathologic results, evaluated semantic CT features in the lung window setting (level, -550 HU; width, 1500 HU) and the mediastinal window setting (level, 40 HU; width, 400 HU). Any disagreements regarding the description of semantic CT features were resolved through consensus reading, and the results were subsequently confirmed by a chief radiologist specializing in chest imaging.

### CT image acquisition and lesion segmentation

Enhanced DICOM CT images were anonymized, and regions of interest (ROIs) were delineated by ITK-SANP software (version 3.8.0; https://www.itksnap.org). According to previous studies [25, 32], the gross tumour volume (GTV) was dilated 15 mm in three dimensions and uniformly served as the GTV + PTV (peritumoral volume). The boundaries of the lung nodules were checked by a radiologist and manually adjusted if necessary. Notably, the parts that cross the interlobar pleura, chest wall and mediastinum should be removed [33]. We obtained the PTV by subtracting the two values. To ensure that the PTV did not contain any GTV components, we specifically added 1 mm to the region of the GTV (PTV = GPTV-(GTV + 1 mm)).

To assess the robustness of the intratumoral and peritumoral segmentation methods, 30 patients were randomly selected, and two junior radiologists performed segmentation on their ROIs twice, with a 2-month interval between sessions, to obtain intraclass correlation coefficients (ICCs).

### Radiomic features

The images were resampled using linear interpolation to achieve a uniform voxel size of 1 × 1 × 1 mm^3^ in all three anatomical directions [34], and the image grayscale was discretized to 25 grayscales. We utilized *PyRadiomics* to extract features from segmented GPVs and PTVs [35]. For each region, 14 shape features (3D), 18 first-order features, 24 grey level cooccurrence matrix (GLCM) features, 16 grey level run length matrix (GLRLM) features, 16 grey level size zone matrix (GLSZM) features, 14 grey level dependence matrix (GLDM) features, and 5 neighbouring grey-tone difference matrix (NGTDM) features were obtained. For each GTV region and PTV, 1595 radiomic features were extracted from the images. A detailed list of the extracted features and the parameters used in CT image preprocessing and feature extraction is provided in Appendix E3. All radiomic features extracted were continuous variables. To ensure comparability, we applied Z-score normalization to these features in the same manners as some previous studies [36, 37].

### Feature selection and modelling

Before radiomic feature selection, only reproducible radiomic features with an ICC ≥ 0.8 were included in the analysis [38]. More details are in Appendix E4. Univariate analysis was subsequently performed, and features with a significance level of *P* < 0.01 were retained in the model. Additionally, features with a correlation coefficient exceeding 0.9 were removed. The least absolute shrinkage and selection operator (LASSO) method, which compresses independent variables with little or no influence on 0, was used to select the most robust and nonredundant radiomic features from the extracted features [39].

Clinical parameters were evaluated in combination with selected radiomic features in the multivariable logistic regression model for predicting the presence of OLM (Appendix E5). The radiomics model’s output scores (Rad_score) were merged with the clinical features to construct the nomogram. This comprehensive model effectively integrated both radiomic and clinical parameters, enhancing the overall predictive power and potential clinical utility of the model.

### Radiomics quality score (RQS) evaluation

In line with the imperative for standardization in radiomics research, we conducted an evaluation of our study utilizing the Radiomics Quality Score (RQS), a methodology consistent with previous studies [40, 41].

## Statistical analysis

Continuous variables were compared using two-sample t test, whereas categorical variables were assessed through chi-square or Fisher’s exact tests. The GTV, PTV, GTV + PTV, clinical, and combined models were established and verified by using R (version 4.1.0, https://www.rproject.org). To assess the model’s performance, the area under the ROC curve (AUC) was utilized, with the optimal cut-off value determined using the derived Youden index. Additionally, the model’s accuracy, sensitivity, specificity, negative predictive value, and positive predictive value were computed. Decision curve analysis (DCA) was performed according to the methods of a previous study [42]. The Delong test was used to compare different AUC values [43]. A two-tailed *p* value of less than 0.05 was considered to indicate statistical significance.

## Results

### Patient characteristics

A total of 242 patients (186 men, 56 women) with 242 lesions (OLM-negative, 160; OLM-positive, 82) were included after the application of the exclusion criteria (Fig. 1). The rate of OLM positivity in all patients was 33.9% (82/242). The characteristics of all the patients are detailed in Table 1.

**Table 1:** The parameters in the development of the clinical model

	Training cohort			External validation cohort		
	OLM (-)	OLM (+)	*p* value	OLM (-)	OLM (+)	*p* value
	*N* = 98	*N* = 60		*N* = 62	*N* = 22	
Gender			0.052			0.546
Female	18 (18.367%)	20 (33.333%)		12 (19.355%)	6 (27.273%)	
Male	80 (81.633%)	40 (66.667%)		50 (80.645%)	16 (72.727%)	
Age	63.000 [57.000;68.000]	61.500 [55.000;66.500]	0.204	62.500 [56.000;67.750]	63.000 [57.000;67.000]	0.579
Smoke			0.002*			0.012
No	24 (24.490%)	30 (50.000%)		14 (22.581%)	12 (54.545%)	
Yes	74 (75.510%)	30 (50.000%)		48 (77.419%)	10 (45.455%)	
Family history			0.655			0.371
No	81 (82.653%)	52 (86.667%)		58 (93.548%)	19 (86.364%)	
Yes	17 (17.347%)	8 (13.333%)		4 (6.452%)	3 (13.636%)	
Lobe			0.753			0.472
RUL	27 (27.551%)	12 (20.000%)		17 (27.419%)	4 (18.182%)	
RML	6 (6.122%)	3 (5.000%)		1 (1.613%)	1 (4.545%)	
RLL	19 (19.388%)	16 (26.667%)		13 (20.968%)	4 (18.182%)	
LUL	27 (27.551%)	16 (26.667%)		15 (24.194%)	9 (40.909%)	
LLL	19 (19.388%)	13 (21.667%)		16 (25.806%)	4 (18.182%)	
Location			0.023*			0.071
Center	20 (20.408%)	23 (38.333%)		6 (9.677%)	6 (27.273%)	
Peripheral	78 (79.592%)	37 (61.667%)		56 (90.323%)	16 (72.727%)	
Clinical stage T			0.051			0.085
1	61 (62.245%)	27 (45.000%)		33 (53.226%)	17 (77.273%)	
2	37 (37.755%)	33 (55.000%)		29 (46.774%)	5 (22.727%)	
Shape			0.033*			0.677
Irregular	67 (68.367%)	30 (50.000%)		47 (75.806%)	15 (68.182%)	
Round or oval	31 (31.633%)	30 (50.000%)		15 (24.194%)	7 (31.818%)	
Branching			0.423			0.280
No	83 (84.694%)	47 (78.333%)		60 (96.774%)	20 (90.909%)	
Yes	15 (15.306%)	13 (21.667%)		2 (3.226%)	2 (9.091%)	
Lobulation			0.581			0.506
No	9 (9.184%)	8 (13.333%)		9 (14.516%)	5 (22.727%)	
Yes	89 (90.816%)	52 (86.667%)		53 (85.484%)	17 (77.273%)	
Spiculation sign			0.501			1.000
No	69 (70.408%)	46 (76.667%)		45 (72.581%)	16 (72.727%)	
Yes	29 (29.592%)	14 (23.333%)		17 (27.419%)	6 (27.273%)	
Calcification			0.635			1.000
No	96 (97.959%)	58 (96.667%)		60 (96.774%)	22 (100.000%)	
Yes	2 (2.041%)	2 (3.333%)		2 (3.226%)	0 (0.000%)	
Concavity			1.000			.
No	97 (98.980%)	59 (98.333%)		62 (100.000%)	22 (100.000%)	
Yes	1 (1.020%)	1 (1.667%)		0 (0.000%)	0 (0.000%)	
Carcinoma			0.527			1.000
No	41 (41.837%)	29 (48.333%)		27 (43.548%)	10 (45.455%)	
Yes	57 (58.163%)	31 (51.667%)		35 (56.452%)	12 (54.545%)	
Bronchial			0.053			0.345
No	64 (65.306%)	29 (48.333%)		40 (64.516%)	11 (50.000%)	
Yes	34 (34.694%)	31 (51.667%)		22 (35.484%)	11 (50.000%)	
Air-bronchogram			0.302			.
No	94 (95.918%)	55 (91.667%)		62 (100.000%)	22 (100.000%)	
Yes	4 (4.082%)	5 (8.333%)		0 (0.000%)	0 (0.000%)	
Obstructive			0.022*			0.770
No	74 (75.510%)	34 (56.667%)		48 (77.419%)	18 (81.818%)	
Yes	24 (24.490%)	26 (43.333%)		14 (22.581%)	4 (18.182%)	
Enhancement Heterogeneity			0.015*			0.112
homogeneous	27 (27.551%)	6 (10.000%)		9 (14.516%)	7 (31.818%)	
not homogeneous	71 (72.449%)	54 (90.000%)		53 (85.484%)	15 (68.182%)	
BVB			0.951			0.761
No	57 (58.163%)	36 (60.000%)		41 (66.129%)	16 (72.727%)	
Yes	41 (41.837%)	24 (40.000%)		21 (33.871%)	6 (27.273%)	
Pleural Retraction			0.800			0.720
No	86 (87.755%)	51 (85.000%)		53 (85.484%)	20 (90.909%)	
Yes	12 (12.245%)	9 (15.000%)		9 (14.516%)	2 (9.091%)	
Pleural Attachment			0.069			1.000
No	75 (76.531%)	37 (61.667%)		46 (74.194%)	17 (77.273%)	
Yes	23 (23.469%)	23 (38.333%)		16 (25.806%)	5 (22.727%)	
Peripheral Emphysema			0.124			0.469
No	57 (58.163%)	43 (71.667%)		32 (51.613%)	14 (63.636%)	
Yes	41 (41.837%)	17 (28.333%)		30 (48.387%)	8 (36.364%)	
Interstitial Pneumonia			0.749			0.053
No	92 (93.878%)	55 (91.667%)		61 (98.387%)	19 (86.364%)	
Yes	6 (6.122%)	5 (8.333%)		1 (1.613%)	3 (13.636%)	

*Significant difference (*p* < 0.05). RUL, right upper lung; RML, right middle lung; RLL, right lower lung; LUL, left upper lung; LLL, left lower lung; BVB, bronchovascular bundle thickening

Clinical parameters that were significant at *p* ≤ 0.05 in univariate analysis were subsequently entered into multivariate analysis. Furthermore, smoking status and shape (*P* < 0.05) were also included in the multivariate analysis.

### Feature selection and model construction

**Fig. 2:**
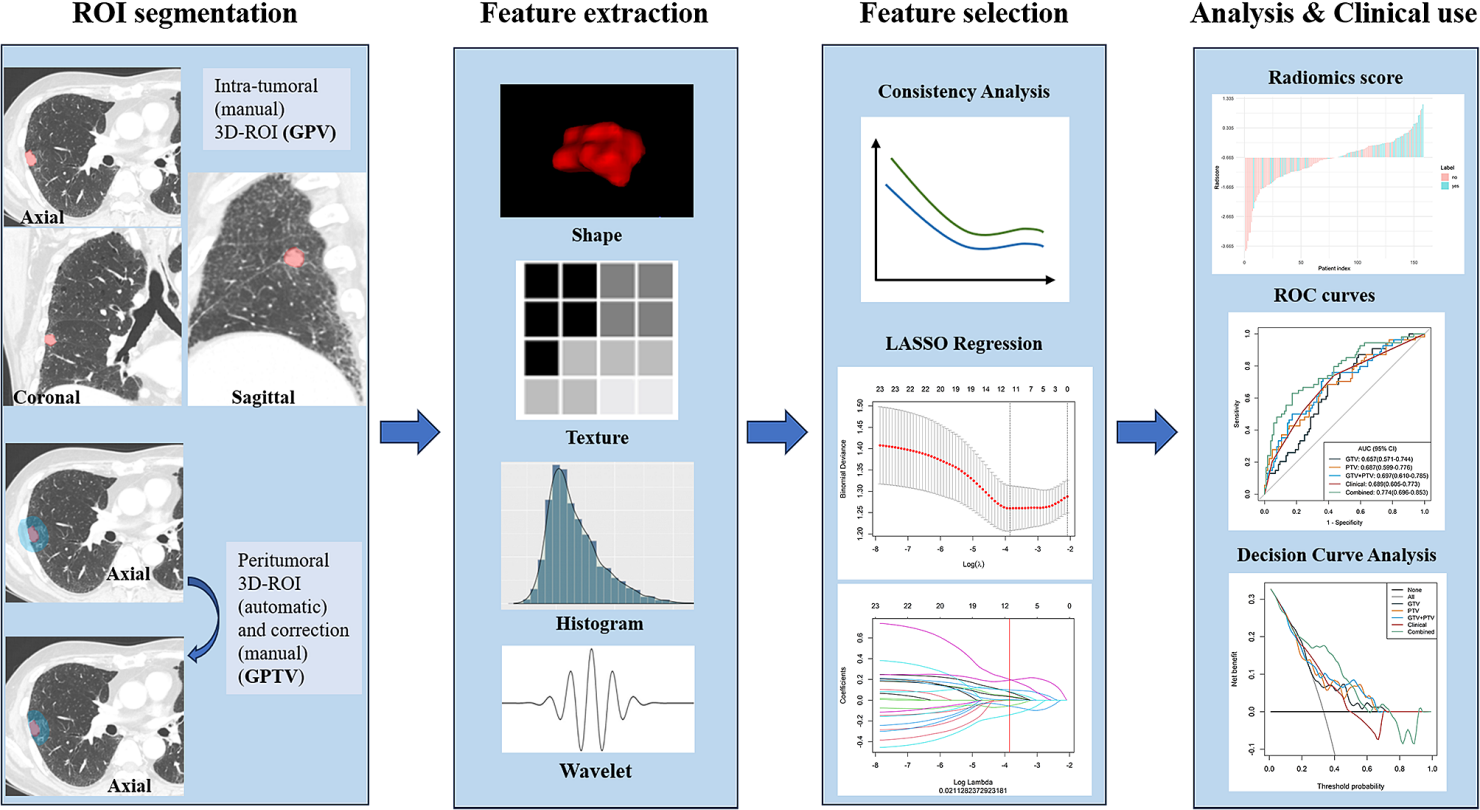
Workflow of radiomic analysis

Figure 2 shows the workflow of the radiomic feature analysis. The radiomic features were selected by using the ICC, univariate analysis, multivariate analysis, correlation analysis, LASSO regression, and multivariable logistic regression. Finally, two radiomic features were selected and utilized to construct the GTV model, and three radiomic features were used to construct the PTV model. After the five selected features were integrated, correlation analysis and multivariate stepwise regression were performed, resulting in the final selection of three features—i.e., the MCC from the GTV, median and IDN from the PTV—for use in constructing the GTV + PTV model. The combined model was established by incorporating one GTV radiomic feature (MCC), two PTV radiomic features (median and IDN), and two clinical parameters. The parameters of the five models are detailed in Appendix E6.

### Performance and comparison of the three models for all patients

All five models have some predictive power. The AUC values of the combined model were 0.774 and 0.772 in the training and external testing cohorts, respectively, performing better than any other models in our study.

**Fig. 3:**
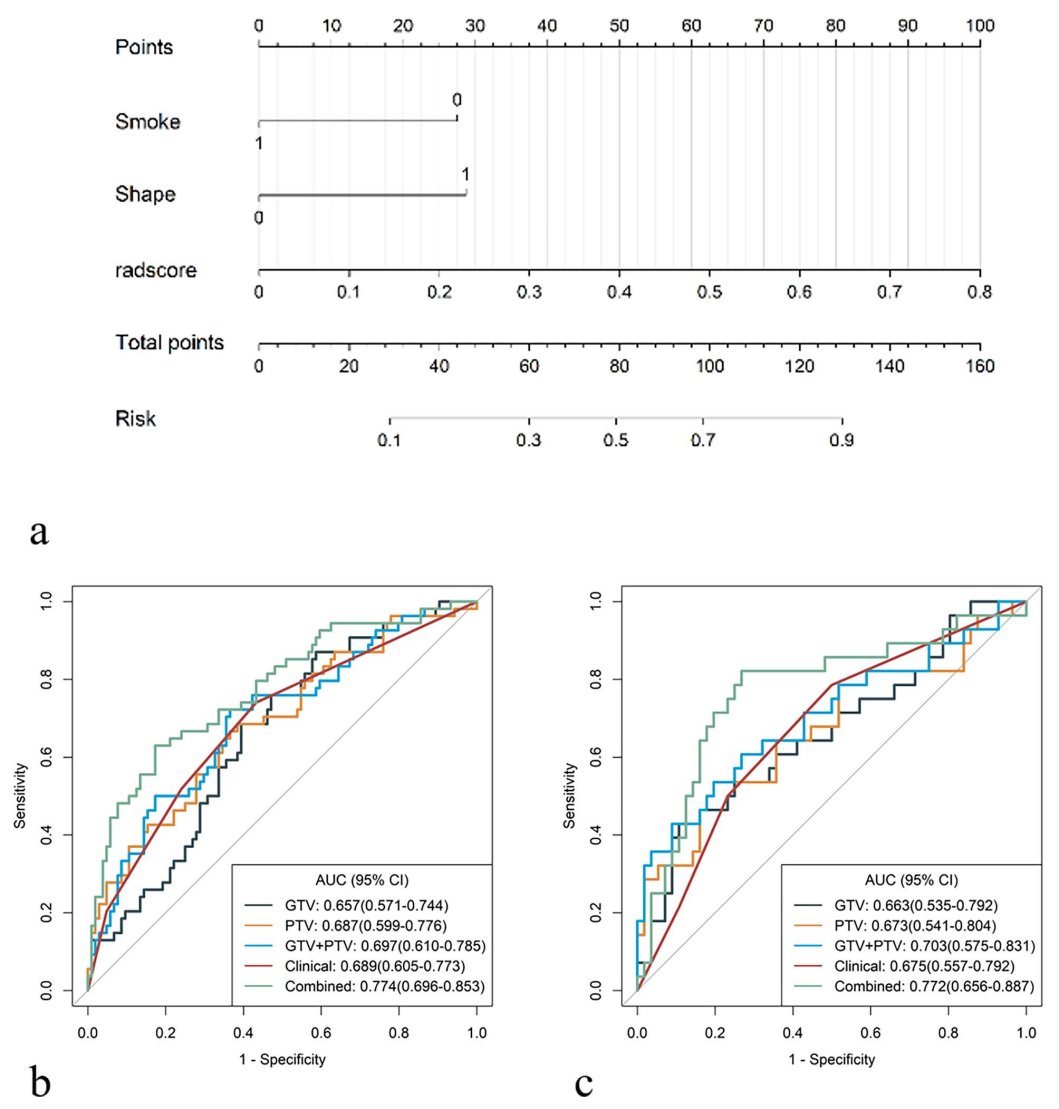
Demonstration of the radiomic nomogram and ROC curves. (**a**) A radiomic nomogram incorporating clinical parameters, GTV, and PTV features was constructed. (**b, c**) ROC curves showing the performance of the GTV model, PTV model, GTV + PTV model, clinical model, and combined model for the prediction of OLM in the training (**b**) and external validation (**c**) cohorts

**Fig. 4:**
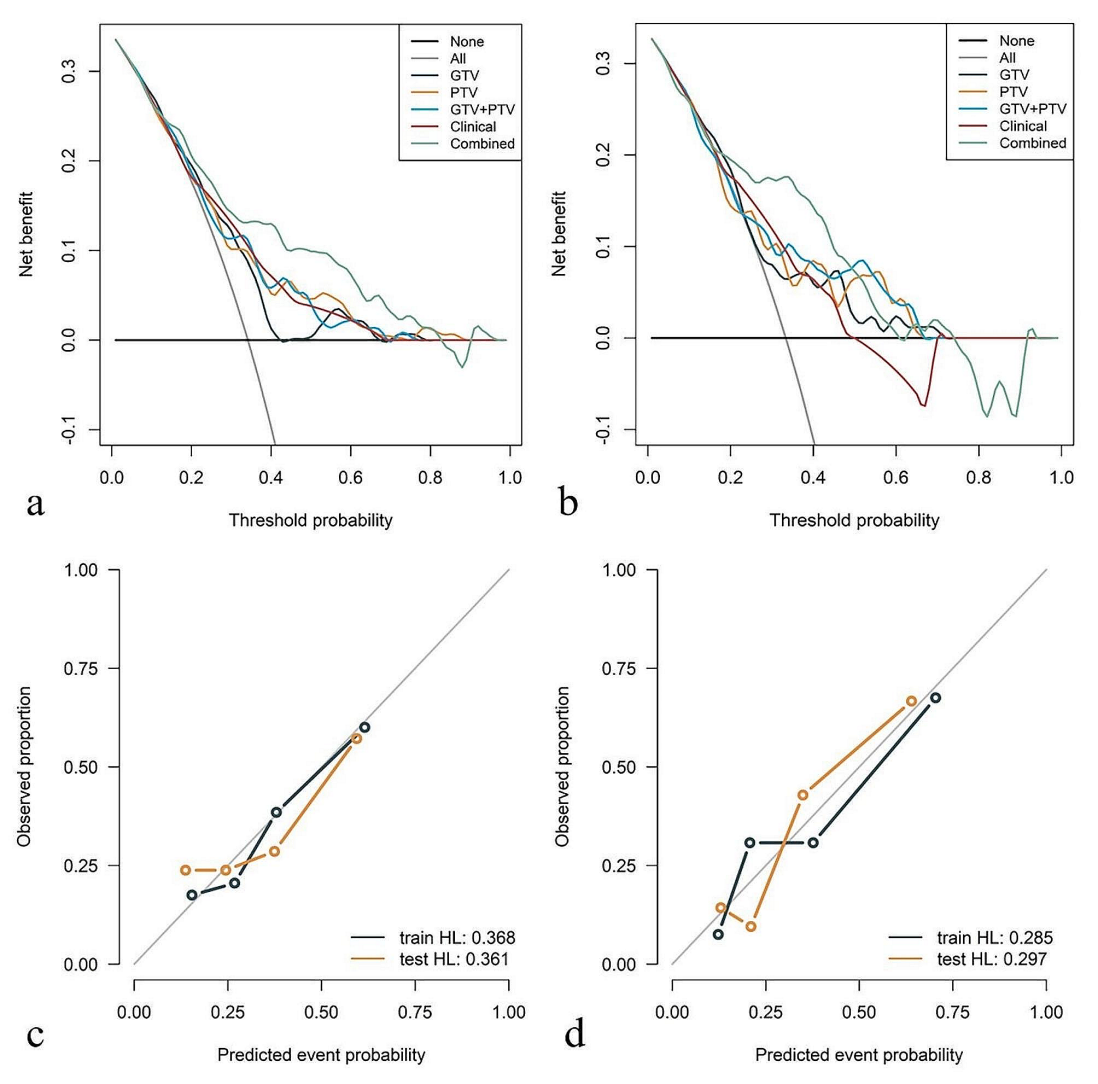
Decision curve analysis of the training cohort (**a**) and external validation cohort (**b**). The calibrations of the GTV + PTV model (**c**) and combined model (**d**)

All the results regarding predictive performance are enumerated in Table 2, and the ROC curves are shown in Fig. 3. The correlation analysis of clinical and radiomic features is indicated in Appendix E5. With respect to the training cohort, the DeLong test revealed significant differences in the area under the curve (AUC) (*p* < 0.05) between the GTV model and the combined model, between the PTV model and combined model, between the GTV + PTV model and combined model, and between the clinical model and combined model. With respect to the external testing cohort, the Delong test revealed that there were significant differences in the area under the curve (AUC) between the GTV + PTV model and the combined model (*p* < 0.05) (Appendix E7). The DCAs (Fig. 4) revealed that when the probability of the threshold was between approximately 10 ~ 80%, the net benefits of the combined model and the GTV + PTV model for the prediction of OLM were greater than those of any other type of model. The calibration plot revealed good predictive accuracy between the actual probability and the predicted probability of the GTV + PTV model and the combined model (Fig. 4).

**Table 2:** Performance of the five models

	AUC (95% CI)	ACC	SEN	SPE	PPV	NPV
Training cohort (*n* = 158)						
GTV	0.657(0.571–0.744)	0.633	0.685	0.606	0.474	0.788
PTV	0.687(0.599–0.776)	0.646	0.667	0.635	0.486	0.786
GTV + PTV	0.697(0.610–0.785)	0.665	0.722	0.635	0.506	0.815
Clinical	0.689(0.605–0.773)	0.627	0.741	0.567	0.471	0.808
Combined	0.774(0.696–0.853)	0.759	0.63	0.827	0.654	0.811
External test cohort (*n* = 84)						
GTV	0.663(0.535–0.792)	0.607	0.607	0.607	0.436	0.756
PTV	0.673(0.541–0.804)	0.607	0.536	0.643	0.429	0.735
GTV + PTV	0.703(0.575–0.831)	0.667	0.607	0.696	0.5	0.78
Clinical	0.675(0.557–0.792)	0.595	0.786	0.5	0.44	0.824
Combined	0.772(0.656–0.887)	0.762	0.679	0.804	0.633	0.833

### RQS evaluation

The total RQS score was 36, and our study obtained a score of 24 (24/36, 66.7%) (Appendix E8).

## Discussion

In this multicenter study, for the first time, we revealed a 33.9% positivity rate for OLM among patients with cT_1 − 2_N_0_M_0_ SCLC. This observation suggested that OLM in cT_1 − 2_N_0_M_0_ SCLC surpasses the prevalence observed in NSCLC, where it ranges from 16 to 29% [26–29, 44–50]. A recent multicenter study of solid-predominantly invasive lung adenocarcinoma had an OLM metastasis rate of 36.1% [24]. In addition, we addressed a crucial challenge in managing cT_1 ~ 2_N_0_M_0_ SCLC by developing and validating predictive models for OLM. Our combined model consistently outperformed the other models in our study, as evidenced by the higher area under the curve (AUC) values in both the training cohort (0.774) and the validation cohort (0.772). According to the model, patients identified as having a higher risk of OLM in cT_1 − 2_N_0_M_0_ SCLC could avoid unnecessary surgeries. Conversely, individuals assessed as having a lower risk might be more confidently considered for surgical resection, with the potential for significant improvements in survival. This study offers a promising approach for accurately identifying OLM in cT_1 − 2_N_0_M_0_ SCLC patients, guiding personalized treatment decisions.

In terms of clinical parameters and conventional CT features, smoking status and tumour shape exhibited noteworthy differences in predicting OLM status, while the remaining features showed no significant distinctions. Our study revealed a tendency for patients with OLM to be smokers, a well-established association with the occurrence and progression of SCLC [12, 51, 52]. In contrast, nonsmokers were more inclined to have OLM in the context of NSCLC [44]. Additionally, we reported for the first time that round and oval tumour shapes hold notable significance, suggesting that lesions with regular shapes may be at a greater risk of OLM positivity. Previous studies on risk factors for NSCLC have been abundant, but uniform results have been lacking, implicating factors such as female sex, adenocarcinoma, density, location and a small tumour size [24, 44, 48, 53, 54]. Thus, there are significant differences in the risk factors for OLM between these two distinct pathological types of lung cancer, enhancing our understanding of OLM in lung cancer patients. However, further studies with larger patient cohorts are necessary to validate our findings.

Following our in-depth analysis, features with ICC values equal to or greater than 0.8 were specifically chosen during feature preprocessing, emphasizing their reliability and repeatability. After a rigorous feature selection process, three radiomic features were ultimately selected: one was the MCC according to intratumoral imaging, and the other two were the median and IDN according to peritumoral imaging. The MCC and IDN are obtained from the grey level co-occurrence matrix (GLCM), which is a texture analysis method that describes spatial relationships between neighbouring pixels to reflect the internal texture of tumours, such as the complexity and heterogeneity of the tumour regions and peritumor regions [55]. The first-order median is expressed as the median grey-level intensity of all pixels in the ROI, which can reflect the textural characteristics of regions around the lesions. Based on our results, even if the imaging findings may be similar in both groups, the MCC, median, and IDN in OLM may serve as noninvasive predictive biomarkers and provide additional information from both intra- and peritumor radiomic data. Then, the selection of an appropriate method for creating combined predictions after variable selection in our study was a deliberate choice aimed at maximizing the predictive power and clinical interpretability of our model. It is widely used by scholars to create combined predictions using multivariate logistic regression in clinical studies, especially in oncology [25, 56–58]. Its broad applicability is one of its considerable advantages. And nomograms are universally recognized for their ability to offer individualized risk assessments presented in a user-friendly graphical format visually [59, 60]. Numerous previous studies have focused on the application of nomograms in depicting OLM [21, 23, 56]. The nomogram’s simplicity and interpretability make it a valuable tool in a clinical setting. Combining intra‑ and peritumoral radiomics with clinical variables in a nomogram allows for the integration of both radiomic and clinical information, leveraging the strengths of each domain.

Surgical intervention has emerged as a highly impactful therapeutic modality for cT_1 − 2_N_0_M_0_ SCLC, emphasizing the crucial role of promoting this approach in clinical practice [61]. A critical consideration is determining whether patients lacking observable lymph node enlargement on routine imaging harbour OLMs. In clinical practice, CT serves as the primary method for preoperative lymph node staging in patients with lung cancer, commonly using a short-axis diameter greater than 1 cm as a threshold [44]. However, OLM cannot be assessed. PET-CT supplements this assessment but has inherent limitations, including false positives and negatives [62], and its high cost makes widespread clinical application challenging [44]. This study presented a comprehensive noninvasive model that demonstrated good performance across all dimensions, boasting a specificity of 82.7% and a sensitivity of 63% on the training dataset. The model exhibited robustness during external validation with a specificity of 80.4% and a sensitivity of 67.9%. A comparison of the five models using DCA curves revealed that our integrated model outperformed the others within the 10-80% probability threshold range. Within this clinically relevant range, both the specificity and sensitivity were considered acceptable. The effectiveness of all the models underscores the inadequacy of traditional methods for evaluating our study’s specific objectives, establishing a significant advantage in efficacy for our research in the field. These findings hold substantial clinical relevance for cT_1 − 2_N_0_M_0_ SCLC patients identified with preoperative negative OLMs, emphasizing the potential impact of timely surgical intervention. The comparison of AUC values between radiomic models utilized the pairwise DeLong test, with corresponding *p* values provided in Supplement E7. In the training cohort, four *p* values derived from the DeLong test were less than 0.05, suggesting that the combination of the GTV and PTV radiomic features with clinical parameters may surpass the performance of a single radiomic feature. Notably, clinical parameters play a pivotal role in predicting OLM in patients with cT_1 − 2_N_0_M_0_ SCLC. In the validation cohort, only the comparison between the GTV model and the combined model yielded a *p* value less than 0.05, implying that the combined model has a greater predictive ability than the solitary GTV model. Additionally, for the first time, we employed radiomics for prediction, revealing its pioneering significance. Future enhancements with increased data volume will further boost the model’s efficacy.

Moreover, our research offers notable advantages. First, this study pioneers the application of radiomic techniques for OLM prediction in cT_1 − 2_N_0_M_0_ SCLC patients, advancing clinical diagnostic proficiency and facilitating precise decision-making and tailored treatment. Second, as a multicenter study, this study included a substantial sample size within the realm of enhanced CT-based radiomic research. Third, the combined model consistently demonstrated stable and commendable performance across both the internal training and external validation datasets, while the nomogram provided visualization and served as a valuable clinical tool for predicting OLM in presurgical cT_1 − 2_N_0_M_0_ SCLC patients. Lastly, The RQS has been widely recognized as a valuable tool for assessing the quality of radiomics studies [40]. The total RQS score for our study was 24 (24/36, 66.67%), surpassing the scores of most radiomics studies [63, 64]. This high score indicates the scientific rigor and reproducibility of our research. While our study received commendable ratings overall, consistent with the standard practice of rigorous methodological validation, there are areas that require improvement. These include the incorporation of biological correlates and the integration of genetic sequencing, both of which necessitate additional funding and patient enrollment. To comprehensively address these aspects, we intend to increase our investment in future research endeavors.

Our study has certain limitations. Firstly, selection bias is inherent in retrospective studies and is exacerbated by a modest sample size. Secondly, in the validation cohort, only the GTV model versus combined model comparison had a *p*-value less than 0.05. Although the combined model performed better in the training cohort, its generalizability to the validation cohort may be limited due to demographic variations across centers. Larger sample sizes in future studies are needed to address this limitation. Thirdly, diverse machine parameters across different hospitals may introduce variations. Nevertheless, this variability contributes to the robustness of the models we trained. Lastly, compared to traditional radiomic methods, deep learning enhances the prediction model’s performance to some extent. Emerging machine learning technologies such as convolutional neural networks are particularly suitable for classification tasks. Our future studies will prioritize data from larger sample sizes and incorporate deep learning applications to further enhance the robustness and performance of our models [65–67].

In conclusion, OLM is not rare and has a greater incidence than NSCLC. Our combined model, which incorporates both intra- and peritumoral radiomic features based on contrast-enhanced CT imaging, serves as a valuable tool for discerning OLM in cT_1 − 2_N_0_M_0_ SCLC patients, guiding individualized clinical decisions.

## Electronic supplementary material

Below is the link to the electronic supplementary material.

Supplementary Material 1

## Data Availability

The datasets used and/or analysed during the current study are available from the corresponding author on reasonable request.

## Abbreviations

SCLC: Small cell lung cancer
NSCLC: Non-small cell lung cancer
OLM: Occult lymph node metastases
CT: Computed tomography
GTV: Gross tumour volume
PTV: Peritumoural volume
GPTV: Gross tumour volume
ICC: Intraclass correlation coefficient
LASSO: Least absolute shrinkage and selection operator
AUC: Area under curve
RQS: Radiomics quality score
