# Supplementary material for: Incidence rate of occult lymph node metastasis in clinical T1−2N0M0 small cell lung cancer patients and radiomic prediction based on contrast-enhanced CT imaging: a multicenter study: Original research
Source: Respir Res. 2024 May 29;25:226. doi: 10.1186/s12931-024-02852-9 (PMC11138070; doi:10.1186/s12931-024-02852-9)

**Supplementary Materials**

**Appendix E1** **The CT protocols of the four centers**

|  | | |  |  |
| --- | --- | --- | --- | --- |
| Parameters | Center 1 | Center 2 | Center 3 | Center 4 |
| Hospital | National Cancer Center/National Clinical Research Center for Cancer/Cancer Hospital | The First Hospital of China Medical University | Shanxi Cancer Hospital | Sun Yat-sen University Cancer Center |
| CT version | Optima CT660, BrightSpeed CT, Revolution CT, Discovery CT750 (GE Medical System, Milwaukee, WI) and Toshiba Aquilion 64-slice spiral CT | Siemens (SOMATOM Force, a dual-source CT scanner with 192 layers), GE (Discovery CT 750 HD, a multilayer CT system with 64 layers), Toshiba (Aquilion One, a scanner with 320 layers), and Philips (Brilliance iCT, a scanner with 256 layers). | Spectral CT (Discovery CT750 HD scanner, GE Healthcare, USA) or a Siemens SOMATOM Force scanner (Siemens Healthineers, Germany | SOMATOM Force (Siemens Healthineers, Forchheim, Germany), Discovery CT750 HD (GE Healthcare, Chicago, IL), Brilliance iCT (Philips, Best, Netherlands), and Aquilion- 64 (Toshiba Medical Systems Corporation, Otawara, Japan) |
| CT tube voltage | 120 kVp | 120 kVp | 120 kVp | 120 kVp |
| CT tube current | 200–350 mAs | 100-200 mA | 375 mA | Auto |
| Contrast agent type | Iopromide | Omnipaque | Omnipaque | Omnipaque |
| Contrast agent dosage | 80 to 90 ml | 70~90 mL | infused 1.5 ml/kg body weight | 1.3 mL/kg of body weight, total of 60-110 mL |
| Contrast agent infused rate | 2.5 to 3.0 ml/s | 2.5~3.0 mL/s | 3.5 ml/s | 2.5 mL/s |
| Image matrix | 512 × 512 | 512 × 512 | 512×512 | 512 × 512 |
| Reconstruction Image thickness | 0.625-1.25mm | 1-1.25mm | 0.625-1.25mm | 1mm |
| Reconstruction kernel | Soft tissue algorithm | Soft tissue algorithm | Soft tissue algorithm | Soft tissue algorithm |
| Field of view (mm) | 350-400 | 350-400 | 350-400 | 350-400 |

**Appendix E2 The definition and evaluation criteria of clinical parameters**

Gender (male or female), Age, smoke (yes or no), Family history (yes or no), Lobe, Location (peripheral or central), clinical-stage T (1 or 2), Shape (round/oval, irregular), Branching (yes or no), Lobulation (yes or no), Spiculation sign (yes or no), Calcification (yes or no), Concavity (yes or no), Carcinoma (yes or no), Bronchial (yes or no), Air-bronchogram (yes or no), Obstructive (yes or no), Enhancement Heterogeneity (yes or no), BVB (yes or no), Pleural Retraction (yes or no), Pleural Attachment (yes or no), Peripheral Emphysema (yes or no), Interstitial Pneumonia (yes or no).

**Appendix E3**

**E3-1 Extraction settings**

The radiomic features with detailed extraction had the following settings: the bin size was 25, voxel shift (minimum value in HU is -1000, shift +1000 to prevent negative values from being squared) was 300, and the voxel size was 1 mm isotropic (using linear interpolation method), applied only on the original image.

**E3-2 Source extraction**

- Original images (morphology + histogram + texture, 107 features)
- Laplacian of Gaussian filtered images (LoG σ=1, 3, 5; histogram + texture, 93 × 3 = 279 features)
- Wavelet transformed images (8 wavelet filters, histogram + texture, 93 × 8 = 744 features)
- Logarithmically filtered images (Logarithm, histogram + texture, 93 features)
- Gradient filtered images (Gradient, histogram + texture, 93 features)
- Local Binary Pattern filtered images (LBP, 3 filter parameters, histogram + texture, 93 × 8 = 279 features)

**E3-3 Features details**

| Shape features | Voxel Number, Volume, Surface Area, Surface Area to Volume ratio, Sphericity, Maximum 3D diameter, Maximum 2D diameter (Slice), Maximum 2D diameter (Column), Maximum 2D diameter (Row), Major Axis, Minor Axis, Least Axis, Elongation, Flatness. | | | |
| --- | --- | --- | --- | --- |
| First-order statistical features | Energy, Total Energy, Entropy, Minimum intensity value, 10th percentile intensity value, 90th percentile intensity value, Maximum intensity value, Mean intensity value, Median intensity value, Interquartile Range, Range of intensity values, Mean absolute deviation (MAD), Robust Mean Absolute Deviation (rMAD), Root mean square (RMS), Skewness, Kurtosis, Variance and Uniformity. | | | |
| Gray Level Co-occurrence Matrix (GLCM) features | Autocorrelation, Joint Average, Cluster Prominence, Cluster Shade, Cluster Tendency, Contrast, Correlation, Difference Average,  Difference Entropy,  Difference Variance, Joint Energy,  Joint Entropy,  Informational Measure of Correlation (IMC) 1,  Informational Measure of Correlation (IMC) 2, Inverse Difference Moment (IDM),  Inverse Difference Moment Normalized (IDMN),  Inverse Difference (ID),  Inverse Difference Normalized (IDN),  Inverse Variance,  Maximum Probability, Sum Average,  Sum Entropy, and Sum of Squares. | | | |
| Gray-level size zone matrix (GLSZM) based features | Small Area Emphasis (SAE), Large Area Emphasis (LAE), Gray Level Non-Uniformity (GLN), Gray Level Non-Uniformity Normalized (GLNN),  Size-Zone Non-Uniformity (SZN), Size-Zone Non-Uniformity Normalized (SZNN),  Zone Percentage (ZP), Gray Level Variance (GLV),  Zone Variance (ZV),  Zone Entropy (ZE),  Low Gray Level Zone Emphasis (LGLZE), High Gray Level Zone Emphasis (HGLZE), Small Area Low Gray Level Emphasis (SALGLE),  Small Area High Gray Level Emphasis (SAHGLE),  Large Area Low Gray Level Emphasis (LALGLE), and Large Area High Gray Level Emphasis (LAHGLE). | | | |
| Gray Level Run Length Matrix (GLRLM) features | Short Run Emphasis (SRE), Long Run Emphasis (LRE), Gray Level Non-Uniformity (GLN),  Gray Level Non-Uniformity Normalized (GLNN),  Run Length Non-Uniformity (RLN), Run Length Non-Uniformity Normalized (RLNN), Run Percentage (RP),  Gray Level Variance (GLV), Run Variance (RV),  Run Entropy (RE),  Low Gray Level Run Emphasis (LGLRE), High Gray Level Run Emphasis (HGLRE),  Short Run Low Gray Level Emphasis (SRLGLE), Short Run High Gray Level Emphasis (SRHGLE), Long Run Low Gray Level Emphasis (LRLGLE), and Long Run High Gray Level Emphasis (LRHGLE). | | | |
| Neigbouring Gray Tone Difference Matrix (NGTDM) features | Busyness, Coarseness, Complexity, Contrast, and Strength. |  |  |  |
| Gray Level Dependence Matrix (GLDM) features | Small Dependence Emphasis (SDE),  Large Dependence Emphasis (LDE), Gray Level Non-Uniformity (GLN), Dependence Non-Uniformity (DN), Dependence Non-Uniformity Normalized (DNN),  Gray Level Variance (GLV), Dependence Variance (DV), Dependence Entropy (DE), Low Gray Level Emphasis (LGLE), High Gray Level Emphasis (HGLE), Small Dependence Low Gray Level Emphasis (SDLGLE),  Small Dependence High Gray Level Emphasis (SDHGLE), Large Dependence Low Gray Level Emphasis (LDLGLE), Large Dependence High Gray Level Emphasis (LDHGLE). | | | |
| All radiomic features were normalized (z-score). The feature-extraction algorithm was developed and modified on the basis of an open access program PyRadiomics. | | | | |

**Appendix E4 ICC analysis**

**GTV ICC Analysis Results:**

Total number of initial features: 1595

Features remaining after ICC: 1245

Features removed: 262

**PTV ICC Analysis Results:**

Total number of initial features: 1595

Features remaining after ICC: 906

Features removed: 601

These results outline the feature analysis after Intraclass Correlation Coefficient (ICC) assessment for both GTV and PTV categories. Features were evaluated based on their ICC values and confidence intervals, determining whether they should be retained or removed.

**Appendix E5**

**E5-1**

Final optimal radiomic features were selected separately from the extracted features in both the GTV and PTV categories, followed by the establishment of models for each category using stepwise multivariable methods (GTV model and PTV model). Then, these features were merged from both GTV and PTV categories. Correlation analysis and stepwise multivariable methods were then employed for feature selection and model building (GTV+PTV model), which combines internal solid features with contextual features. Clinical features were subjected to univariate analysis, retaining features with a significance level of p<0.05. Subsequently, a multivariable stepwise regression was employed for feature selection and model construction (Clinical model). The scores generated by the Radiomics model (Rad_score) were combined with clinical features to construct a multivariable logistic regression model (Combined model), thus integrating comprehensive radiomic parameters with clinical parameters.

**E5-2**

GTV model:

After ICC analysis, 1245 group-level features remained; post univariate analysis, 512 features were retained; after correlation analysis, 23 features remained; following LASSO selection, 11 features were retained; ultimately, after multivariable stepwise regression, 2 features were left.

PTV model:

After ICC analysis, 906 group-level features remained; univariate analysis, 296 features were retained; post correlation analysis, 31 features remained; after LASSO selection, 14 features were retained; and after multivariable stepwise regression, 4 features were left.

GTV+PTV model:

Following correlation analysis, the number of features (7) remained unchanged; after multivariable stepwise regression, 3 features were retained.

**Table|** Three radiomic features selected for the combined model

| ROI | Image nature | Filter parameters | Feature type | Feature name |
| --- | --- | --- | --- | --- |
| GTV | Log | 5 | Glcm | MCC |
| PTV | Wavelet | LHL | Firstorder | Median |
| PTV | gradient | - | glcm | Idn |

Clinical model:

Clinical parameters, after univariate analysis and multivariate, Smoke and shape two features were remained.

Combined model:

Two clinical parameters and three radiomic features.

**Appendix E6**

GTV model:

| Parameter | Coef | OR | P |
| --- | --- | --- | --- |
| Cut off value | -0.7533 | - | <0.001 |
| GTV_log.sigma.5.0.mm.3D_glcm_MCC | 0.6794 | 1.973(1.261-3.438) | 0.007 |
| GTV_lbp.3D.m2_ngtdm_Strength | 0.3683 | 1.445(0.998-2.092) | 0.0473 |

GTV Rad_score=-0.7533 + 0.6794* GTV_log.sigma.5.0.mm.3D_glcm_MCC + 0.3683 * GTV _lbp.3D.m2_ngtdm_Strength


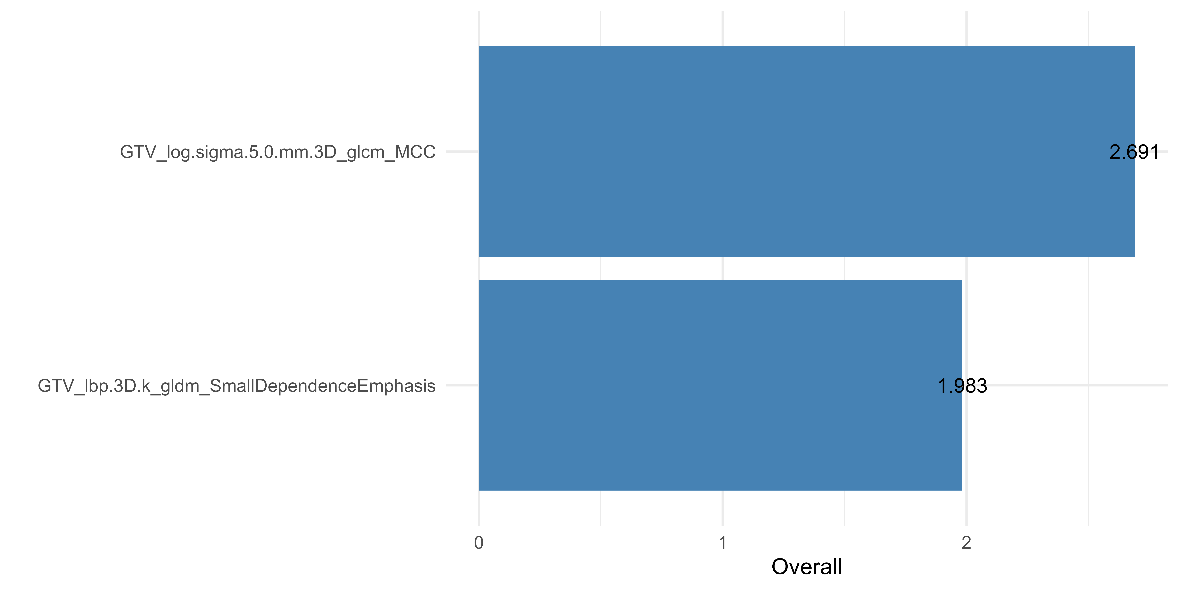


Figure E6-1. The resulting features in the GTV model. The y-axis indicates the selected features, and the x-axis represents the coefficient of features.

PTV model:

| Parameter | Coef | OR | P |
| --- | --- | --- | --- |
| Cut off value | -0.7259 | - | <0.001 |
| PTV_log.sigma.3.0.mm.3D_firstorder_Skewness | 0.5115 | 1.668(1.144-2.525) | 0.011 |
| PTV_wavelet.LHL_firstorder_Median | -0.3497 | 0.705(0.482-1.005) | 0.060 |
| PTV_gradient_glcm_Idn | -0.3975 | 0.672(0.466-0.953) | 0.029 |

PTV Rad_score=-0.7259+0.5115*PTV_log.sigma.3.0.mm.3D_firstorder_Skewness-0.3497*PTV_wavelet.LHL_firstorder_Median–0.3975*PTV_gradient_glcm_Idn


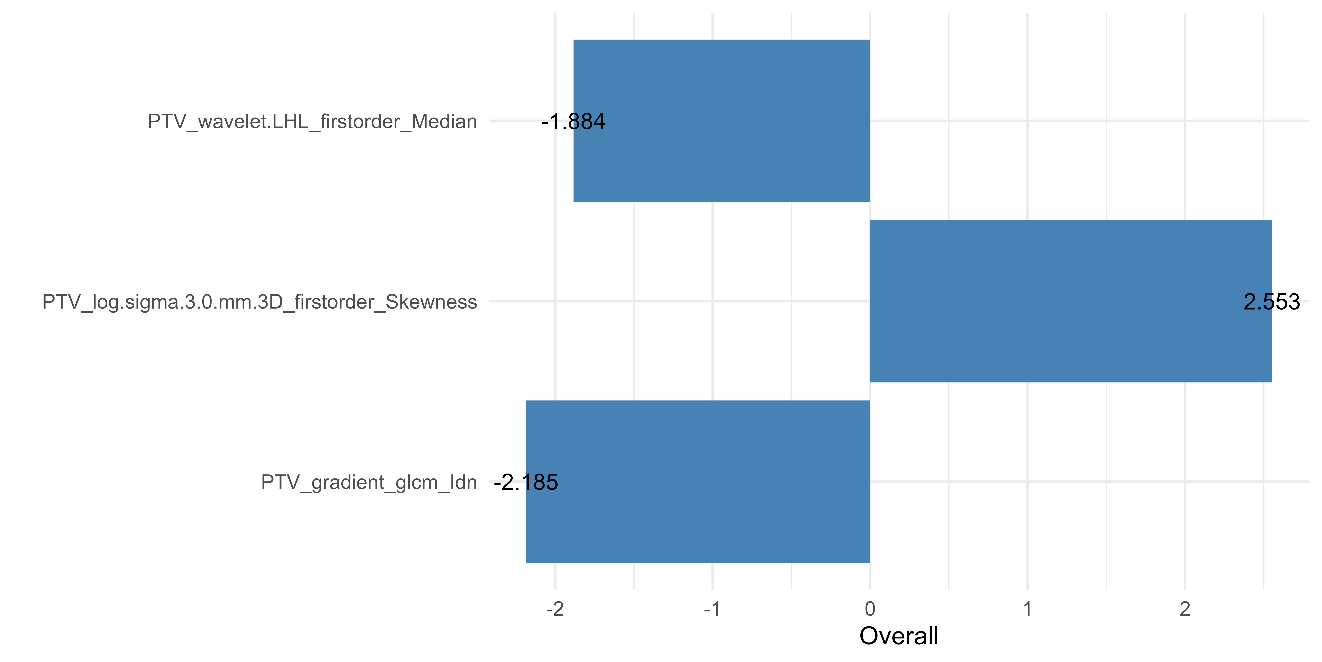


Figure E6-2. The resulting features in the PTV model. The y-axis indicates the selected features, and the x-axis represents the coefficient of features.

GTV+PTV model:

| Parameter | Coef | OR | P |
| --- | --- | --- | --- |
| Cut off value | -0.7706 | - | <0.001 |
| GTV_log.sigma.5.0.mm.3D_glcm_MCC | 0.660 | 1.935(1.199-3.555) | 0.0171 |
| PTV_wavelet.LHL_firstorder_Median | -0.3363 | 0.714(0.489-1.021) | 0.071 |
| PTV_gradient_glcm_Idn | -0.3308 | 0.718(0.500-1.017) | 0.0664 |

GTV+PTV model Rad_score=-0.7706+0.660* GTV_log.sigma.5.0.mm.3D_glcm_MCC-0.3363 PTV_wavelet.LHL_firstorder_Median-0.3308 PTV_gradient_glcm_Idn


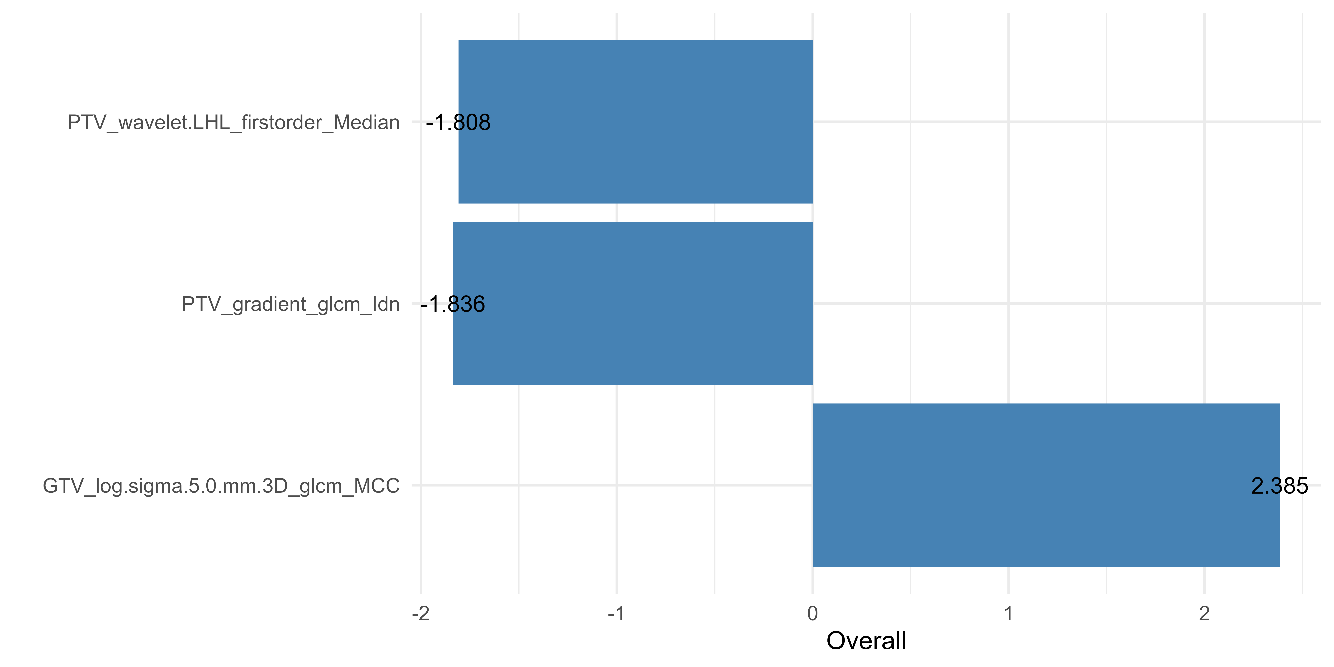


Figure E6-3. The resulting features in the GTV+PTV model. The y-axis indicates the selected features, and the x-axis represents the coefficient of features.

Clinical model:

| Parameter | Coef | OR | P |
| --- | --- | --- | --- |
| Cut off value | -0.1584 | - | 0.596 |
| Smoke | -1.2834 | 0.277(0.133-0.546) | <0.001 |
| Shape | 0.9360 | 2.55(1.221-5.399) | 0.0131 |

Clinical model Rad_score=-0.1584-1.2834*Smoke+0.9360*Shape


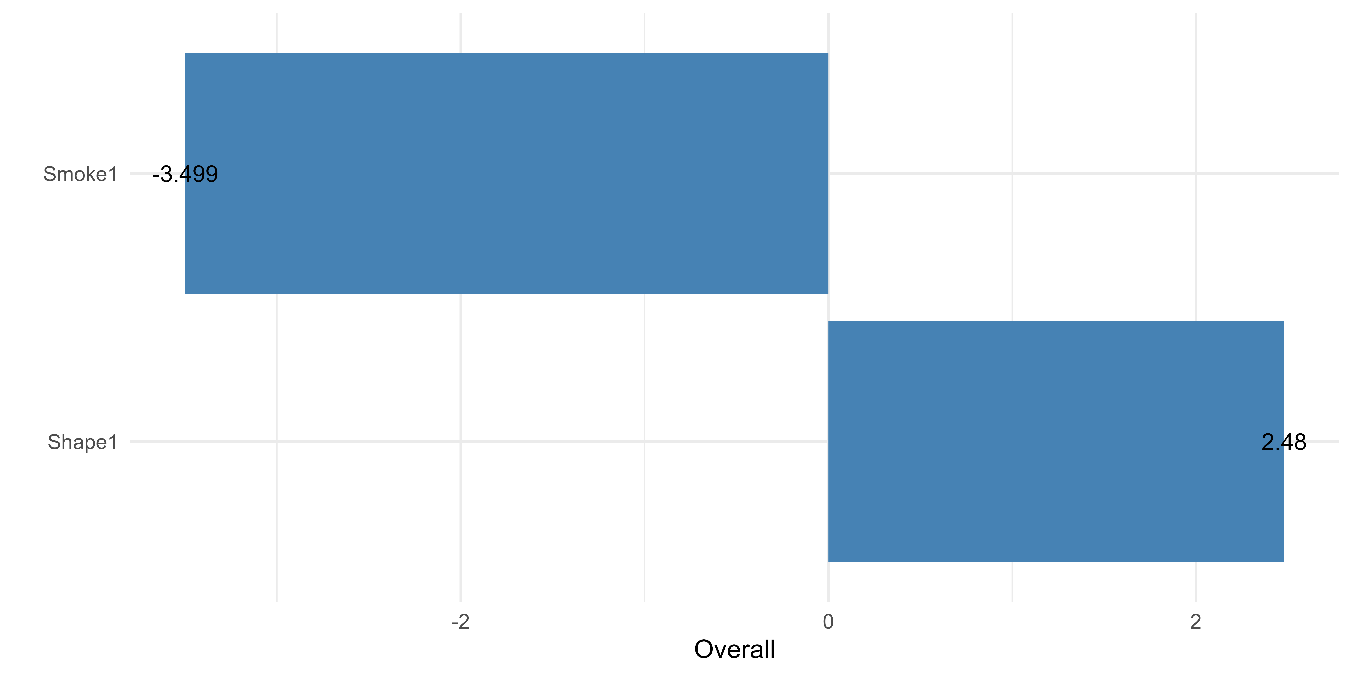


Figure E6-4. The resulting features in the clinical model. The y-axis indicates the selected features, and the x-axis represents the coefficient of features.

Combined model:

| Parameter | Coef | OR | P |
| --- | --- | --- | --- |
| Cut off value | -2.264 | - | <0.001 |
| Smoke | -1.2044 | 0.3(0.137-0.641) | 0.002 |
| Shape | 1.2557 | 3.51(1.557-8.259) | 0.003 |
| GPTV Rad_score | 5.4736 | 238.324(17.14-4409.79) | <0.001 |

Combined model Rad_score=-2.264-1.2044* Smoke + 1.2557*Shape+5.4736*GPTV Rad_score


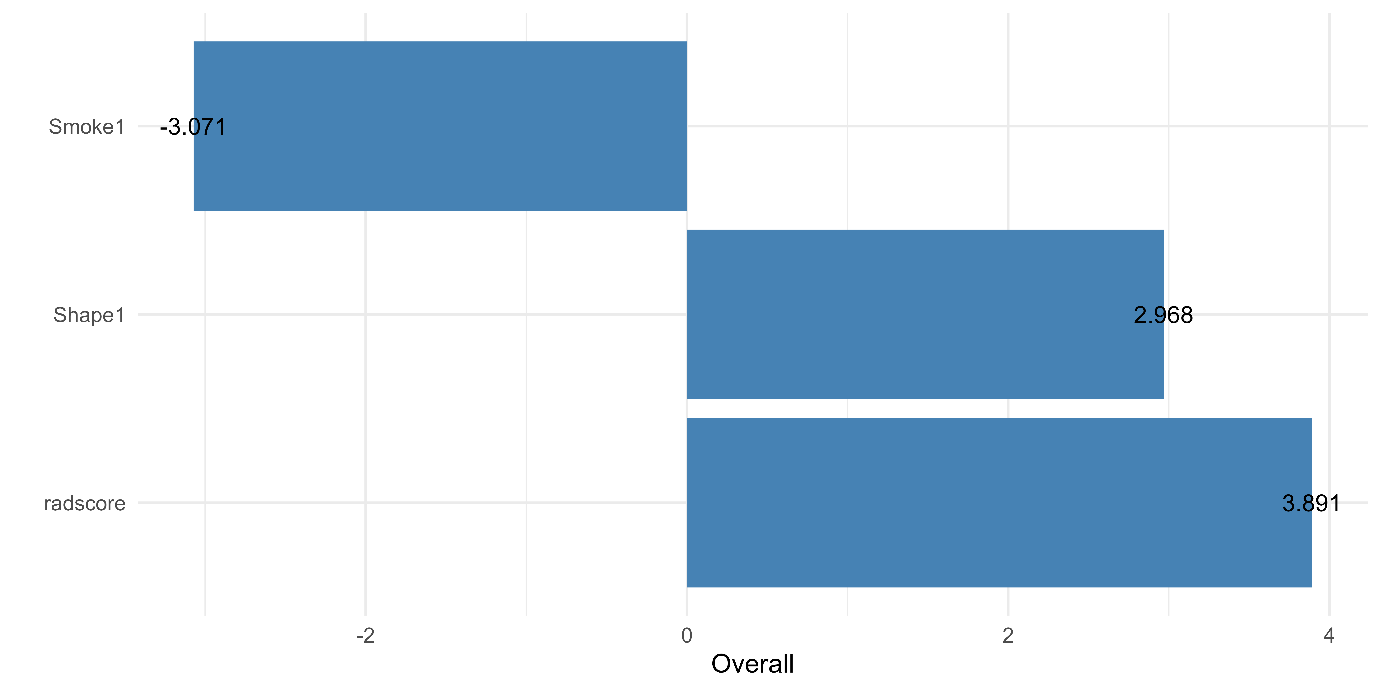


Figure E6-5. The resulting features in the combined model. The y-axis indicates the selected features, and the x-axis represents the coefficient of features.

**Appendix E7** **P value from the Delong test on the training and validation cohorts**

**Training cohort**

| Name | p_value | z_value |
| --- | --- | --- |
| GTV model -PTV model | 0.478837 | -0.70817 |
| GTV model -GTV+PTV model | 0.182584 | -1.33284 |
| GTV model -Clinical model | 0.596251 | -0.5298 |
| GTV model -Combined model | 0.004859* | -2.81623 |
| PTV model -GTV+PTV model | 0.753811 | -0.31362 |
| PTV model -Clinical model | 0.975567 | -0.03063 |
| PTV model -Combined model | 0.034666* | -2.11224 |
| GTV+PTV model -Clinical model | 0.8929 | 0.134636 |
| GTV+PTV model – Combined model | 0.03619* | -2.09479 |
| Clinical model – Combined model | 0.011091* | -2.53983 |

*Significant difference (p < 0.05)

**External validation cohort**

| Name | p_value | z_value |
| --- | --- | --- |
| GTV model -PTV model | 0.852012542 | -0.18655 |
| GTV model -GTV+PTV model | 0.257644435 | -1.13198 |
| GTV model -Clinical model | 0.892823115 | -0.13473 |
| GTV model -Combined model | 0.03843035* | -2.07023 |
| PTV model -GTV+PTV model | 0.476550175 | -0.71186 |
| PTV model -Clinical model | 0.983534926 | -0.02064 |
| PTV model -Combined model | 0.121362063 | -1.54908 |
| GTV+PTV model -Clinical model | 0.752638513 | 0.315162 |
| GTV+PTV model – Combined model | 0.167450534 | -1.38044 |
| Clinical model – Combined model | 0.07366289 | -1.7887 |

*Significant difference (p < 0.05)

**Appendix E8** **Radiomics Quality Score (RQS)**


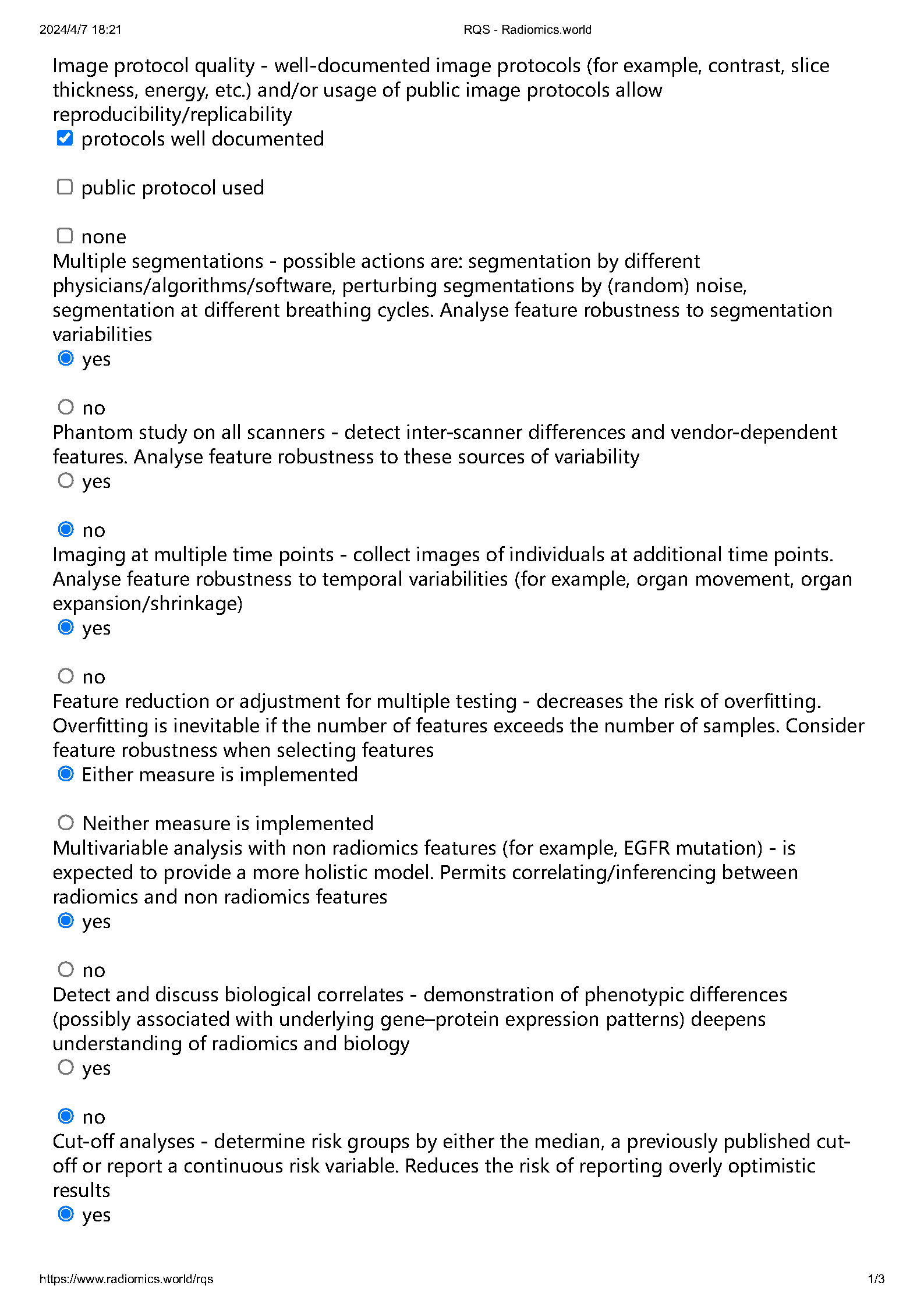


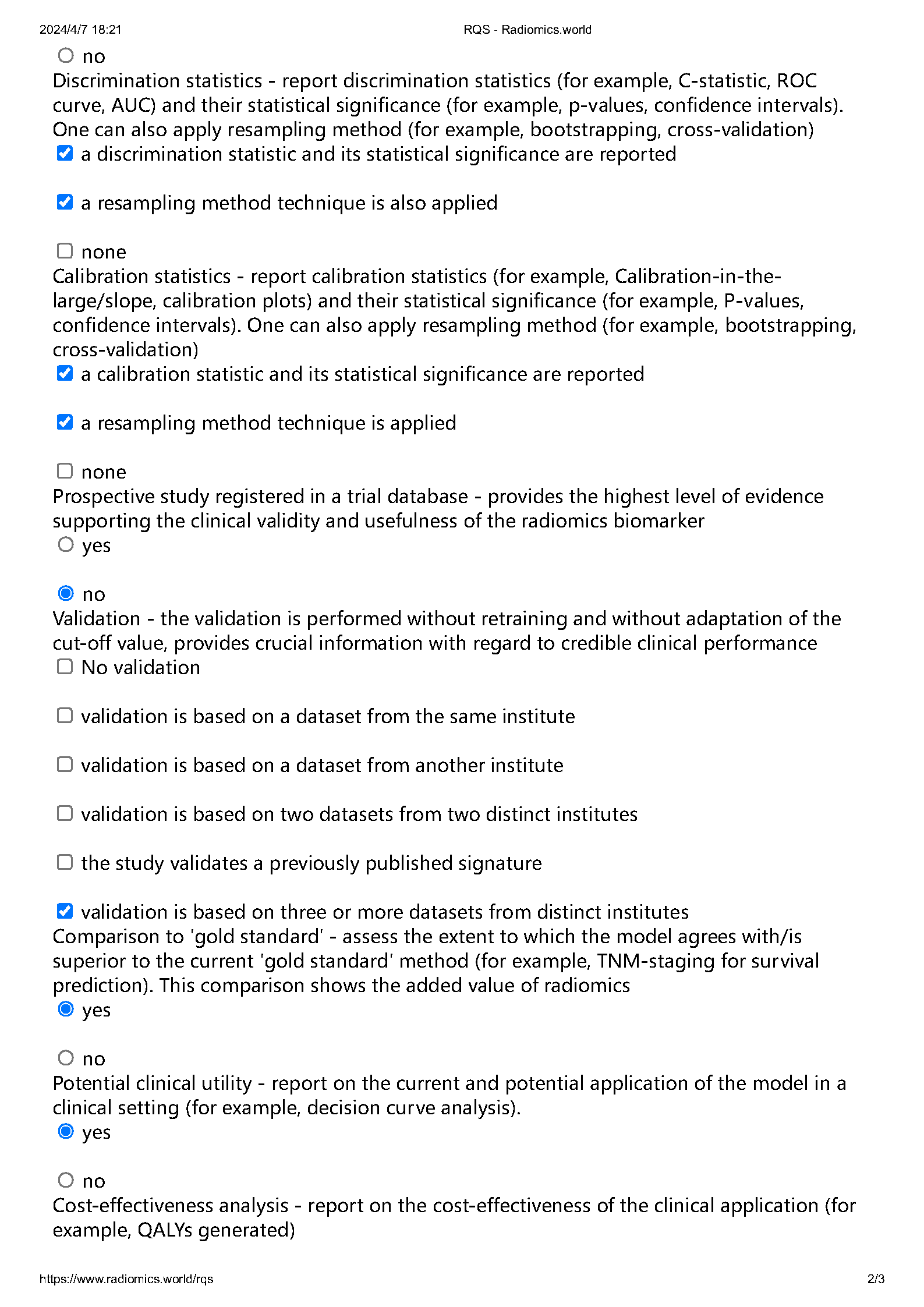


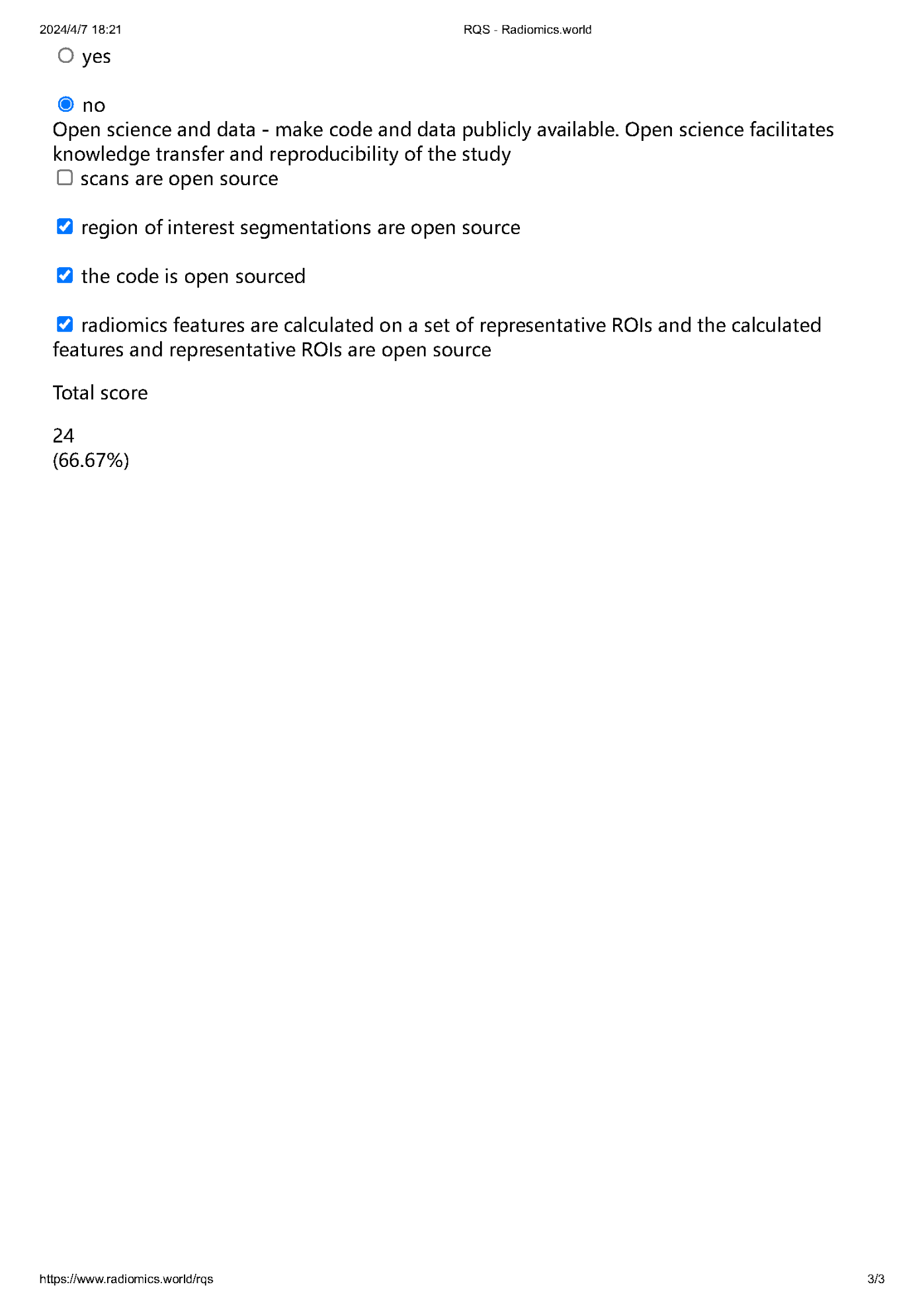

Supplement: Supplementary file 1 — Supplementary Material 1 [file 12931_2024_2852_MOESM1_ESM.docx]
